# Village malaria worker performance key to the elimination of artemisinin-resistant malaria: a Western Cambodia health system assessment

**DOI:** 10.1186/s12936-016-1322-6

**Published:** 2016-05-20

**Authors:** Sara E. Canavati, Saranath Lawpoolsri, Cesia E. Quintero, Chea Nguon, Po Ly, Sasithon Pukrittayakamee, David Sintasath, Pratap Singhasivanon, Koen Peeters Grietens, Maxine Anne Whittaker

**Affiliations:** Department of Clinical Tropical Medicine, Faculty of Tropical Medicine, Mahidol University, 420/6 Ratchawithi Road, Ratchathewi, Bangkok, 10400 Thailand; Centre for Biomedical Research, Burnet Institute, Melbourne, Australia; Department of Tropical Hygiene, Faculty of Tropical Medicine, Mahidol University, 420/6 Ratchawithi Road, Ratchathewi, Bangkok, 10400 Thailand; The National Center For Parasitology, Entomology and Malaria Control, Ministry of Health, Corner Street 92, Trapaing Svay Village, Sankat Phnom Penh Thmey, Khan Sensok, Phnom Penh, Cambodia; Mahidol-Oxford Tropical Medicine Research Unit, Faculty of Tropical Medicine, Mahidol University, 420/6 Ratchawithi Road, Ratchathewi, Bangkok, 10400 Thailand; Malaria Consortium Asia, Faculty of Tropical Medicine, Mahidol University, 420/6 Rajavidhi Road, Room 805, Bangkok, 10400 Thailand; Medical Anthropology Unit, Department of Public Health, Institute of Tropical Medicine, Antwerp, Belgium; School of Tropical Medicine and Global Health, Nagasaki University, Nagasaki, Japan; Partners for Applied Social Sciences (PASS) International, Tessenderlo, Belgium; Division of Tropical Health and Medicine, College of Public Health, Medical and Veterinary Sciences, James Cook University, Townsville, QLD 4811 Australia; The University of Queensland School of Public Health, Herston, QLD 4006 Australia

**Keywords:** Community malaria worker, Artemisinin resistance, Cambodia, Malaria elimination, Health system strengthening

## Abstract

**Background:**

Village malaria workers (VMWs) and mobile malaria workers (MMWs) are a critical component of Cambodia’s national strategy to eliminate *Plasmodium falciparum* malaria by 2025. Since 2004, VMWs have been providing malaria diagnosis through the use of rapid diagnostic tests and free-of-charge artemisinin-based combination therapy in villages more than 5 km away from the closest health facility. They have also played a key role in the delivery of behaviour change communication interventions to this target population. This study aimed to assess the job performance of VMWs/MMWs, and identify challenges they face, which may impede elimination efforts.

**Methods:**

A mixed-methods assessment was conducted in five provinces of western Cambodia. One hundred and eighty five VMW/MMW participants were surveyed using a structured questionnaire. Qualitative data was gathered through a total of 60 focus group discussions and 65 in-depth interviews. Data triangulation of the qualitative and quantitative data was used during analysis.

**Results:**

Overall, VMWs/MMWs met or exceeded the expected performance levels (80 %). Nevertheless, some performance gaps were identified. Misconceptions regarding malaria transmission and prevention were found among workers. The recommended approach for malaria treatment, directly-observed treatment (DOT), had low implementation rates. Stock-outs, difficulties in reaching out to migrant and mobile populations, insufficient means of transportation and dwindling worker satisfaction also affected job performance.

**Discussion:**

VMW/MMW job performance must be increased from 80 to 100 % in order to achieve elimination. In order to do this, it is recommended for the national malaria programme to eliminate worker malaria knowledge gaps. Barriers to DOT implementation and health system failures also need to be addressed. The VMW programme should be expanded on several fronts in order to tackle remaining performance gaps. Findings from this evaluation are useful to inform the planning of future activities of the programme and to improve the effectiveness of interventions in a context where artemisinin drug resistance is a significant public health issue.

## Background

Improving access to diagnosis and treatment and providing appropriate health education for at-risk populations are essential components of successful malaria control [1]. Access to early detection and treatment can be improved through the deployment of community health workers. Since the Alma Ata Declaration in 1978, international efforts to improve access to primary health care have shifted towards the deployment of various types of community health workers (CHWs). Worldwide, malaria community-based interventions by CHWs have successfully improved health outcomes and decreased malaria mortality in resource-poor settings [2–4].

CHWs can play a crucial role in addressing health workforce shortages, especially in rural and remote areas of the country [2], as has been the case for Cambodia. Amongst the ten countries that comprise the Association of Southeast Asian Nations (ASEAN), Cambodia has the greatest sub-national inequities in its distribution of medical doctors [5]. Most public health facilities in Cambodia are under-staffed, or lack health providers who can deliver appropriate services in rural and remote areas [6]. Village-based volunteer workers have played an important role in malaria diagnosis and treatment in many different settings for more than 35 years. Two of these programmes stand out in terms of their size and longevity: the Volunteer Collaborator Network of Latin America [7] and the Village Voluntary Malaria Collaborator Programme of Thailand [8]. The success of these programmes has been attributed to the cultural tradition of community participation and sustained commitment already present in these regions, as well as to support from national malaria control programmes. Small, established rural communities in Cambodia also have a strong tradition of community participation in political decision-making, both among ethnic minorities [9] and in the general population, save for a brief disruption during the Khmer Rouge era [10, 11].

Following a pilot phase, the village malaria worker (VMW) project was launched in 2004 as a National Programme for Parasitology, Entomology and Malaria Control (CNM)-led scheme [12]. It provided essential supplies for VMWs to access communities in remote villages. Within the then-labelled “containment zones”, VMWs targeted the general population, whereas mobile malaria workers (MMWs) targeted the migrant population. At that point in time, a high level of poor health indicators in target communities justified this focused and vertical approach. By 2008, 400 of the most malaria-endemic communities in Cambodia were being provided early malaria diagnosis and treatment through the village-based volunteer network. The project was initially seen as a medium-term venture that would be scaled down in accordance with the gradual expansion and strengthening of static health services. Nevertheless, it was later expanded to provide a surveillance and education function, which has become integral to Cambodia’s malaria elimination efforts.

**Fig. 1:**
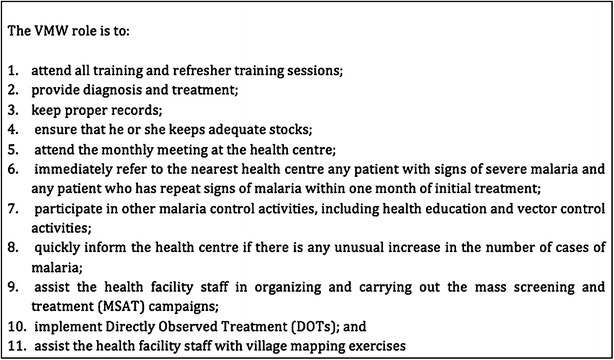
VMW roles and responsibilities

Under the Bill & Melinda Gates Foundation-funded and World Health Organization (WHO)-led (2007–2009) strategy for containment of artemisinin-resistant *Plasmodium falciparum* parasites in South-East Asia [13], the CNM and other partners endeavoured to contain the spread of artemisinin-resistant (ART-R) *P. falciparum* parasites. Recently, Cambodia set itself the ambitious goal of eliminating falciparum malaria by 2025 [14]. At present, this elimination effort is focused on those provinces in which ART-R elimination zones are located. Within the containment project, the VMW project was significantly expanded in ART-R zones 1 and 2. Over a period of only 2 years, the number of VMW-assisted villages was increased three-fold, from 400 to 1123 villages. In these containment zones, VMW responsibilities had a strong focus on Directly Observed Therapy (DOT), screening, and treatment (Fig. 1).

## Methods

In order to evaluate the quality and scope of VMW activities within the containment project zones, as well as the challenges VMWs/MMWs face, a mixed methods assessment was conducted in five Western Cambodian provinces. The objectives of this study were to: (i) appraise VMW and MMW job satisfaction; (ii) assess their performance according to their job descriptions; and (iii) gain insight into the challenges inherent in delivering diagnosis, treatment and health education activities at the community level.

### Qualitative strand

#### Data collection and sampling

The qualitative assessment was conducted in five provinces, including 36 villages and eight health centres (HCs). Qualitative data were gathered through a total of 60 focus group discussions (FGDs) (416 participants) and 65 in-depth interviews (IDIs) conducted between October 2011 and January 2012. The semi-structured part of the qualitative data collection instruments (i.e. FGD and IDI guides) were initially developed based on the authors’ previous work [15] and on the health belief model [16], which was further adapted for the purpose of this study.

FGD and IDI participants included VMWs and MMWs, public health facility staff, village chiefs, villagers, migrants, and malaria patients. Purposive sampling was used to select participants to ensure gender distribution, a variety of ages, geographical provenances, intra-provincial travel capacity, migration statuses and occupations.

#### Focus group discussions

A total of 36 village-based FGDs were conducted in VMW-assisted villages. For (i) FGDs with *community members*, two villages were purposively selected for geographical balance per HC. As male and female community members have different risks and vulnerabilities (e.g. tendency for forest work), in each village two male and two female FGDs were conducted. Each village-based FGD consisted of eight participants (n = 32). (ii) Additional male and female FGDs were conducted with *migrant workers*, consisting of eight participants (n = 16). (iii) FGDs with *VMWs/MMWs* were also conducted. In order to have sufficient participants for the farm-based FGDs, VMWs/MMWs were selected, based on operational district (OD) rather than HC. Each VMW/MMW FGD consisted of eight participants (n = 16); two male and two female FGDs were conducted per OD.

#### In-depth interviews

Selection criteria for all IDIs was for participants to: (1) have been a resident of the community for at least 2 years, in order to ensure that they had been exposed to the VMW programme for some period of time; (2) be between 25–45 years of age, as this age range is most exposed to malaria in the study site [17–20].

For both FDGs and IDIs, *community members, migrant workers,* and *the village chief* were interviewed in each selected village. In each health centre, the *HC staff*, *VMWs*, *MMWs* were interviewed.

#### Data analysis

FDGs and IDIs were recorded, fully transcribed, and translated into English. Data analysis consisted of examining, categorizing, tabulating or recombining the data, in order to address the study’s initial aim. Thematic analysis around the key themes of the project was undertaken using Nvivo 9^®^ software; transcripts were read and re-read for themes that emerged from the data. These themes were clustered to form overarching, larger themes. Triangulation and critical case analysis added rigour to the process.

### Quantitative strand

#### Data collection and sampling

A cross-sectional study was carried out, using a structured questionnaire. The sample size was calculated based on an expected 80 % VMW/MMW job performance rate, with a 5 % acceptable error. With a total of 534 VMWs and 108 MMWs in the five selected provinces, a type I error of 0.05, and a 10 % estimated non-response rate, it was calculated that a total of 196 VMWs and MMWs were required for the study.

Subsequently, five ODs within the five provinces were randomly selected and stratified by zone (two ODs from Zone 1 and three ODs from Zone 2). Within these ODs, all HCs with 10 or more VMWs or MMWs were selected as potential sites for sample recruitment. All VMWs and MMWs within those HCs were asked to participate in the study, as per exhaustive sampling. About 95 % of those VMWs and MMWs were included in the study; the other 5 % did not attend the survey.

#### Data analysis

Data were double-entered using an Epi Info^®^ database. Analysis was performed using Stata^®^ version 11 (StataCorp LP, College Station, TX, USA). Descriptive statistics, including basic frequencies and simple proportions, were calculated. The Mantel–Haenszel Chi square test or the Fisher’s exact test was used to calculate significance.

### Ethical issues

Ethical clearance was obtained from the Cambodian National Ethics Committee for Health Research in August 2011 (130NECHR) and from the Ethics Committee of the Faculty of Tropical Medicine of Mahidol University (MUTM 2012-021-01). Local authorities (village leader and commune chief) and community leaders were informed of the purpose and expected duration of the study.

For the qualitative strand, the interviewers followed the Code of Ethics of the American Anthropological Association (AAA). As proposed by the AAA, all interviewees were informed before the start of the interview about project’s goals, the topic and type of questions, the intended use of results for scientific publications, and their right to refuse the interview, interrupt the conversation at any time, and withdraw all given information during or after the interview. Anonymity was guaranteed and the confidentiality of interviewees assured by assigning a unique code number to each informant. Written consent was obtained. Each survey participant provided informed written consent before participation. Participant’s approval and cooperation was sought in every aspect of data collection. A mid-term data collection meeting was conducted in Phnom Penh, where the team leaders met with the team coordinator to discuss progress and address challenges.

## Results

In the qualitative strand, a total of 65 IDIs were conducted among farm owners, MMWs, VMWs, malaria patients, HC staff, community members and village chiefs. From each selected commune in the catchment area, the staff member responsible for coordinating VMW/MMW activities and assessing VMW/MMW performance was also interviewed.

In the cross-sectional quantitative survey, 185 participants responded to the survey (response rate of 94.7 %). The majority were Khmer (98 %), most were married (82 %), and half had completed primary education. Farming was their most common profession (92 %). 58 % were male, and the mean age was 35. Overall, most worker performance indicators were in line with the expected level of performance (80 %), or even higher (Table 1).

**Table 1:** Indicators pertaining to VMWs/MMWs compared with assessment results

Process and outcome indicators	Assessment results
% of contact points (based on initial situational analysis) providing malaria diagnosis, treatment, prevention, and messages to mobile/migrant populations	Most VMWs and MMWs reported working with mobile and migrant groups: 179/197 = 91.0 %
All VMWs and MMWS are fully trained according to plan by 06/09	The majority of VMWs and MMWs received training during the year 2009: 178/197 = 90.3 %
% of community level staff (VMW/MMW) are aware of key messages	Seek treatment from VMW or HC: 167/197 = 88.36 % Sleep under an ITN: 161/197 = 85.2 % Sleep under a net when visiting the forest: 132/197 = 69.8 % Sleep under a net when travelling: 94/197 = 49.7 % Seek treatment within 24 h: 39/197 = 20.6 % Complete anti-malarial treatment: 33/197 = 17.5 % Aware of three key messages: 102/197 = 51.8 %^a^ Aware of four key messages: 63/197 = 32.0 %^b^
% of community level staff (VMW/MMW) who perform according to the TOR	*Findings for the fourth indicator* *% of VMWs and MMWs who perform according to TOR are described below* 1. The VMW shall keep proper records of consultations, diagnosis and treatment on the standard forms *All VMW/MMW reported filling out the monthly list of cases* = 100 % 2. The VMW shall attend the monthly meeting at the health centre where he or she will provide the health care staff with the record forms and pick up a re-supply of RDTs and anti-malarial drugs *Nearly all VMWS and MMWs reported attending the monthly meetings at the health centre on a monthly basis* = 196/197 = 99.5 % 3. The VMW must immediately refer to the nearest health centre any patient with signs of severe malaria and any patient who has repeat signs of malaria within 1 month of initial treatment *It was reported that the main group that gets referred are severe malaria patients* 122/197 = 82.43 %

^a^Three key messages include: seek treatment from VMW or HC; sleep under an ITN; and sleep under a net when visiting the forest

^b^Four key messages include: seek treatment from VMW or HC; sleep under an ITN; sleep under a net when visiting the forest and sleep under a net when travelling

### Malaria knowledge

#### Worker education

The majority (90.0 %) of VMW and MMW participants reported having received the original two-day training in 2009, and refresher training in 2011 (99.5 %). Although more than 33 % of them lived at a considerable distance from the nearest HC, most (99.5 %) said they regularly attended monthly meetings at the HC, as required by their job description. These monthly meetings are part of the continued professional development of the workers, and all the VMWs/MMW interviewed found the monthly meetings useful (Table 1).

**Fig. 2:**
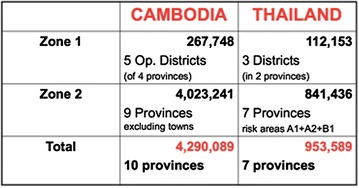
Target populations in the containment project

The respondents were asked about malaria symptoms, treatment and transmission. Symptom recall was high (Table 2), and 99 % of VMWs and MMWs said that a blood test is necessary to confirm malaria diagnoses. 83.3 % of the interviewees understood that not taking the recommended anti-malarial drug regimen can increase parasite resistance. Respondents also recalled that patients who fail to complete treatment regimens can relapse (particularly in Zone 1, P = 0.026), or continue to transmit parasites (particularly in Zone 2, P = 0.051) (Table 3). All respondents knew that malaria is transmitted through mosquito bites. Likewise, risk behaviours, such as sleeping without a mosquito net, and prevention strategies, like wearing protective clothing, had high recall rates. The latter was reported at a statistically significantly higher level in Zone 1 than in Zone 2 (Fig. 2).

**Table 2:** Malaria symptom recall by VMWs and MMWs

Symptoms	% of VMWs/MMWs
Malaria symptoms	
Fever	86.8
Chills	82.2
Headache	79.2
Sweating	75.6
Severe malaria symptoms	
Unconsciousness	83.8
High temperatures	77.7
Convulsions	65.5
Very pale skin	55.8

**Table 3:** Knowledge of malaria reported by VMWs and MMWs by zone

	Overall N = 197		Zone 1 N = 71		Zone 2 N = 126		P value
	n	(%)	n	(%)	n	(%)	
At least three key malaria symptoms^a^	92	46.7	40	56.3	61	48.4	0.521
At least four key malaria symptoms^b^	74	37.6	26	36.6	48	38.1	0.837
Determinants of malaria illness							
Blood test (slide or dipstick)	195	99.0	71	100.0	124	98.4	0.190
Symptoms	57	28.9	19	26.8	38	30.2	0.614
Previous experience	22	11.2	5	7.0	17	13.5	0.286
Doctor’s examination	3	1.5	0	0.0	3	1.5	0.168
Other	4	2.1	1	1.4	3	2.4	0.642
Don’t know	–	0.0	–	0.0	–	0.0	–
At least three key signs and symptoms of severe malaria^c^	88	44.7	35	49.3	53	42.1	0.327
At least four key signs and symptoms of severe malaria^d^	48	24.4	18	25.4	30	23.8	0.809
Ways to get malaria							
Mosquito bites	197	100	71	36.0	126	64.0	–
Sleeping in forest without a net	93	47.2	36	38.7	57	61.3	0.461
No sleep under a mosquito net	91	46.2	31	34.1	60	65.9	0.593
Visiting forest	77	39.1	29	37.7	48	62.3	0.704
Poor hygiene	12	6.1	3	25.0	9	75.0	0.411
Drinking dirty water	9	4.6	5	7.04	4	3.17	0.212
Not boiling water	7	3.6	2	2.8	5	3.9	0.675
Bad food	2	1.0	1	1.4	1	0.8	0.679
Other^e^	3	1.5	0	0.0	3	2.38	0.593
Ways to prevent malaria							
Sleep under a mosquito net	182	92.4	63	88.7	119	94.4	0.147
Use treated mosquito net	180	91.4	68	95.8	112	88.9	0.098
Wear covered clothing	155	78.7	62	87.3	93	73.8	0.026
Burn leaves	32	16.2	9	12.7	23	18.3	0.308
Stay out of forest	15	7.6	7	9.9	8	6.4	0.373
Repellent	5	2.5	2	2.8	3	2.3	0.852
Mosquito coil	2	1.0	2	2.8	0	0.0	0.058
Insecticide spray	1	0.5	9	12.7	23	18.3	0.308
Boil water	41	20.8	12	16.9	29	23.0	0.310
Take medicine	1	0.5	1	1.41	0	0.0	0.182
Other^f^	38	19.3	8	11.27	30	23.8	0.032
Consequences of not taking the anti-malarial drugs for the recommended treatment regimen							
Parasite will become resistant	164	83.3	59	83.1	105	83.3	0.966
Patient gets sick again	155	78.7	62	83.1	93	73.8	0.026
Patient does not recover	93	47.2	31	43.7	62	49.2	0.454
Patient will continue to transmit malaria	78	39.6	26	36.6	52	41.3	0.522
Parasite remains in the body	76	38.6	21	29.6	55	43.7	0.051
Nothing	1	0.51	0	0.0	1	0.8	1.000
Other^a^	7	3.55	0	0.0	7	5.7	0.051

^a^Three key malaria symptoms include: fever, chills, and headache

^b^Four key malaria symptoms include: fever, chills, headache, and sweating

^c^Four key symptoms of severe malaria include: unconscious, very hot, and convulsions

^d^Four key symptoms of severe malaria include: unconscious, very hot, convulsions, and very pale skin

^e^Other include: wearing short sleeves

^f^Other include: clean the surroundings, eliminate mosquito habitat, fill in body of water, light a bonfire, light fire, sanitization, sleep in hammock, use net in the forest, and weeding

Overall, more than 15 % of the answers reflected misconceptions about malaria transmission. Infection sources were sometimes reported to include poor hygiene (6.1 %), dirty drinking water (4.6 %), unboiled water (3.6 %), and bad food (1.0 %). 20.8 % of the total participants deemed boiling water to be a preventive measure (Table 3). This misconception, confirmed by qualitative interviews, was most prevalent in Zone 2. This statement, from an FGD with VMWs, was typical:

*My education methods are: Firstly, I recommend them to drink boiled water. Secondly, to wear long clothing. Thirdly, I ask them not to chat [outdoors] after the sunset. If they want to chat, they must do so under the mosquito nets to prevent malaria. (*FGD15_VMW_Male).

#### Migrant education

The qualitative interviews indicated that migrants were provided with health education comparable to that of the local population. Nevertheless, many VMWs and MMWs discussed how newly-arrived migrants are often unaware of basic preventive measures, and contract malaria shortly after their arrival, before they receive health education. No MMWs, and only one VMW, reported having provided pre-departure malaria education. HC staff supported this observation, strongly recommending that health education be provided to prospective migrants at the pre-departure stage, while they still reside in non-endemic areas. They emphasized that in order to do this, VMW coverage would need to be extended to malaria-free zones.

### Barriers to job performance

The VMW/MMW job description includes eleven activities for which they are held accountable. The qualitative and quantitative strands found significant hindrances to these activities in the following areas:

#### Adherence to DOT guidelines

According to most VMWs and MMWs, they routinely observed the first dose of treatment (98.48 %), in accordance with DOT guidelines. However, DOT on the second and third days of treatment, which is also required by the guidelines, was reportedly adhered to by only 56.8 % of respondents. Both VMWs and MMWs appeared to face similar barriers to DOT implementation. According to the quantitative data, a lack of available transportation was one of the main challenges, as workers must often cover long distances in order to reach patients’ homes. This transportation problem was significantly more likely to be cited as a barrier in Zone 1 (p = 0.000), and was linked to a more frequent reporting of long distances as a problem in Zone 1 (p = 0.001) (Table 4).

**Table 4:** Practices: treatment and DOT

Treatment	Overall N = 197		Zone 1 N = 71		Zone 2 N = 126		P value
	n	(%)	n	(%)	n	(%)	
Place where malaria patients get treated							
At VMW/MMW’s home	193	98.0	71	100.0	122	96.8	0.129
At the patient’s home	156	79.2	61	85.9	95	75.4	0.081
Other^a^	2	1.02	0	0.0	2	1.6	0.286
Malaria patient is directly-observed for the first dose of medication							
Yes	194	98.5	70	98.6	124	98.4	0.693
No	2	1.0	1	1.4	1	0.79	
Sometimes	1	0.5	0	0	1	0.79	
Malaria patient is directly-observed for the 2nd and 3rd days of treatment							
Yes	112	56.9	35	49.3	77	61.1	0.265
No	27	13.7	12	16.9	15	11.9	
Sometimes	58	29.4	24	33.8	34	27.0	
Barriers faced when providing directly-observed treatment							
Problem of transportation to patients’ home	151	76.7	65	91.6	86	68.3	0.000
Long distance to patients home	149	75.6	63	88.73	86	68.3	0.001
Self-treatment	26	13.2	5	7.0	21	16.7	0.055
The patient disagreed	24	12.2	10	14.1	14	11.1	0.540
No knowledge of malaria drug resistance	21	10.7	5	7.0	16	12.7	0.217
Treatment with other providers	3	1.5	0	0.0	3	2.4	0.190
Other^c^	2	1.0	0	0.0	2	1.0	0.286
Don’t know^b^	11	5.6	0	0.0	11	8.7	0.537

^a^Other includes: farm, rice field

^b^Fisher exact test was used to calculate for significance

^c^Other include: death, more severe, waste of time

In addition to their own restricted mobility, VMWs and MMWs reported high patient mobility as a barrier to providing DOT. There was therefore a prevailing perception amongst workers that it is not possible to provide DOT to mobile and migrant populations (MMPs), which was associated with the general agreement that providing malaria education and resources like insecticide-treated mosquito nets to migrants before they migrate is crucial. These perceptions were found in the qualitative interviews, where a typical discussion was:

*We sometimes give migrants malaria drugs to take for 3* *days, but after they take the drugs for only 2* *days they move somewhere else. They might look for drugs in other places. We do not know whether they recovered or not. (*FGD37_VMW_MaLE).

#### Accessibility to patients

HC staff indicated that the VMWs’ geographical proximity to their catchment community significantly increased the feasibility and effectiveness of malaria treatment, particularly when it comes to providing timely outreach education, treatment, and patient follow-up, all of which are pillars of the malaria programme. VMWs were more likely to report being available for consultations in the mornings, and workers were more likely to undertake proactive case finding at that time. In Zone 1, VMW availability for consultations was reported to be significantly higher than in Zone 2. A significantly higher proportion of pregnant women and children under 5 years of age were referred to the HC in Zone 1 as well, and VMWs were more available for consultations (p value 0.011) (Tables 5, 6). VMWs or public health facilities were more likely to provide the message “seek treatment for malaria from a VMW or public health facility” (*P* value = 0.001) in Zone 1 (Table 7).

**Table 5:** Case finding and referrals by zone

Practices	Overall N = 197		Zone 1 N = 71		Zone 2 N = 126		P value
	n	(%)	n	(%)	n	(%)	
Ways in which VMW/MMW find malaria cases in their villages							
Conduct home visits	157	79.7	61	85.9	96	76.2	0.103
Malaria suspects know me and come to my house	154	78.2	52	73.2	102	80.1	0.208
Other people refer them	105	53.3	41	57.8	64	50.8	0.348
Conduct health education activities	100	50.8	39	54.9	61	48.4	0.38
During campaigns conducted by NGOs	2	1.0	0	0.0	2	1.6	0.286
Other	4	2.0	0	0.0	4	3.17	0.129
Refer malaria cases to the health centre or hospital							
Yes	148	75.1	50	70.4	98	77.8	0.404
No	49	24.9	21	29.6	28	22.2	
Who gets referred							
Severe malaria cases	122	82.4	41	82.0	81	82.7	0.921
Pregnant women	47	31.8	21	42.0	26	26.5	0.056
The elderly	44	29.7	15	30.0	29	29.6	0.959
Children under five	37	25.0	17	34.0	20	20.4	0.071
Patients who’s life is at risk	28	18.9	11	22.0	17	17.4	0.494
Other^a^	30	20.3	7	14.0	23	33.5	0.175
Barriers faced when referring severe malaria to the HC							
Lack of transportation	130	87.8	45	90.0	85	86.7	0.565
Long distance	98	66.2	34	68.0	64	65.3	0.743
Lack of money	67	42.3	18	36.0	49	50.0	0.106
The patient disagrees	29	19.6	13	26.0	16	16.3	0.161
Lack of understanding of severity of disease	17	11.5	6	12.0	11	11.2	0.889
Treatment with other providers	10	6.8	2	4.0	8	8.2	0.34
Self-treatment	5	3.4	1	2.0	4	4.1	0.507
Other^b^	4	2.7	0	0.0	4	4.1	0.148

^a^Other includes: patient with multiple illnesses, patient with negative blood test, not malaria cases. Patients also get referred when there is blood test error and VMWs and MMWs medicine is out of stock

^b^Other includes: medicine was out of stock, negative blood test, never have barriers, and never have problems

**Table 6:** General information on consultations by zone

General information on consultations	Total N = 197		Zone 1 N = 71		Zone 2 N = 126		P value
	n	(%)	n	(%)	n	(%)	
Days per week spent on working as a VMW/MMW							
1 day	6	3.05	3	4.2	3	2.4	0.702
2–3 days	138	70.1	51	71.8	87	69.1	
4–6 days	37	18.8	13	18.3	24	19.1	
Whole week	16	8.1	1	5.6	12	9.5	
Daily hours spent working as a VMW/MMW							
1 h	44	22.3	16	22.5	28	22.2	0.242
2–3 h	132	67.0	47	66.2	85	67.5	
4–5 h	16	8.1	8	11.3	8	6.4	
6 or >6 h	5	2.5	0	0.0	5	4.0	
Time of the day available for consultations^a^							
Morning time	170	86.3	67	94.4	103	81.8	0.013
Afternoon time	132	67.0	51	71.8	81	64.3	0.280
Evening	144	73.1	52	73.2	92	73.0	0.973
All day^b^	8	4.1	0	0.0	8	6.4	0.053
Time people come to VMW/MMW for consultation^c^							
Morning time	173	87.8	67	94.4	106	84.1	0.035
Afternoon time	133	67.5	50	70.4	83	65.9	0.513
Evening	143	72.6	52	73.2	91	72.2	0.878
All day	5	2.5	0	0.0	5	4.0	0.089
VMW/MMW sometimes not available for consultations							
Yes	60	30.5	15	21.1	49	38.9	0.011
No	133	67.5	56	78.9	77	61.1	
When VMW/MMW not available for consultations							
Assign a family member/friend to assist VMW	50	78.1	14	99.3	36	73.5	0.391
Refer the patient to another VMW/MMW	6	9.4	0	0.0	6	12.5	
Refer the patient to the health centre	3	4.7	1	6.7	2	4.04	
Ask the patient to come back on the next day	4	6.3	0	0.0	4	8.2	
Other	1	1.6	0	0.0	1	2.0	

^a^Multiple responses possible

^b^Exact fisher test was performed

^c^Multiple responses possible

**Table 7:** Communication and key messages by zone

Communication	Overall N = 197		Zone 1 N = 71		Zone 2 N = 126		P value
	n	(%)	n	(%)	n	(%)	
In the past *1* *month*, VMW/MMW taught any messages or information about malaria							
Yes	189	95.9	70	98.6	119	94.4	0.157
No	8	4.1	1	1.4	7	5.6	
Messages or information related to malaria prevention were taught or explained^a^							
Malaria is caused by mosquito bites	125	64.1	52	74.3	73	61.3	0.069
Wear long sleeved clothes from dusk to dawn to prevent mosquito bites	120	63.5	39	55.7	81	68.1	0.088
Sleep under a mosquito net every night	135	71.4	55	78.6	80	67.2	0.095
Sleep under an insecticide-treated net	161	85.2	60	85.7	101	84.9	0.875
Other^b^	26	14.3	6	8.6	21	17.7	0.085
Messages related to malaria diagnosis and treatment that were taught or explained							
Seek treatment for malaria from a VMW or health facility	167	88.4	69	98.6	98	82.6	0.001
Visit your village malaria worker for free malaria diagnosis and treatment	165	87.3	61	87.1	104	97.4	0.96
Seek treatment for malaria promptly/within 24 h	39	20.6	9	12.9	30	25.2	0.043
Get a blood test before taking anti-malarial drugs	60	31.8	20	28.6	40	33.6	0.472
If you have fever, always seek a blood test for malaria at the nearest health facility	48	25.4	19	27.1	29	24.4	0.672
Complete anti-malarial treatment	33	17.7	7	10.0	26	21.9	0.038
Other	1	0.5	0	0.0	1	0.8	0.442
Messages related to using and caring for mosquito nets that were taught or explained							
Sleeping under an ITN is especially important for pregnant women	62	32.8	27	38.6	35	29.4	0.195
Sleeping under an ITN is especially important for children under 5 years	56	29.6	21	30.0	35	29.4	0.932
Carry and sleep under a mosquito net when travelling	94	49.7	40	57.1	54	45.4	0.118
Carry and sleep under a mosquito net when visiting the forest	132	69.8	49	70.0	83	65.8	0.971
The less you wash your treated net the longer it will retain its effectiveness	119	63.0	40	57.1	79	66.39	0.204
Repair any holes in the net	36	20.0	9	12.9	27	22.7	0.096
Other^c^	62	32.8	22	31.4	40	33.6	0.757
Who did VMW/MMW communicated information about malaria							
Villagers	196	99.5	71	100.0	125	99.21	0.452
Malaria patients	114	57.9	38	53.5	76	60.3	0.354
Migrants/mobile population	158	80.2	60	84.5	98	77.8	0.255
Friends/neighbours	95	48.2	29	40.9	66	52.4	0.12
Family members	100	50.8	32	45.1	68	54.0	0.23
Religious leaders/monks	5	2.5	1	1.41	4	3.2	0.449
Other	3	1.5	1	1.41	2	1.6	0.922
VMW/MMW has distributed or displayed any information about malaria							
Yes	129	65.5	48	67.6	81	64.3	0.638
No	68	34.5	23	32.4	45	35.7	
Kind of information about malaria VMW/MMW displays or distributes							
Poster	90	69.8	34	70.8	56	69.1	0.839
Leaflets/brochures	96	74.4	42	88.0	54	66.7	0.009
Flip charts	27	20.9	5	10.4	22	27.2	0.024
Other^d^	6	4.7	0	0.0	6	7.4	0.053
The last time there was an event in the community that provided malaria-related health messages							
Yesterday	10	5.1	4	5.6	6	4.8	0.371
Within this week	30	15.2	10	14.1	20	15.9	
In this month	105	53.3	43	60.6	62	49.2	
In this year	52	26.4	14	19.7	38	30.1	

^a^Out of the total number of VMW/MMW who have taught any messages or information about malaria

^b^Other include: mosquito habitat elimination, blood testing, clean the surrounding, drink boiled water, eliminate mosquito habitat, fill up pond, light bonfire, know malaria symptoms, use malaria treatment, often clean water containment, use net in forest, weeding, weeding around house

^c^Other include: avoid drying in sunlight, do not sleep close to net, do not use net for fishing, do not wash, do not wash too often, do not wash with detergent, dry net in shade, keep away from children, keep net clean after use, keep net safe, keep net safe after use, must retreat net, put in bag after use, retreat net every 3 months, retreat net every 6 months and wash net every 6 months

^d^Other include: booklet, educating, malaria booklet, malaria prevention, picture calendar, pictures and videos

##### Servicing MMPs appeared to be particularly challenging in both zones

*There are six specific difficulties when working with migrant people. Firstly, migrant people do not have permanent housing. For example, they live under tents in the forest. Secondly, they do not have mosquito nets, and allow themselves to be bitten by mosquitoes. Thirdly, their food is insufficient. Fourthly, they are far away from health services. They also lack means of transportation. Lastly, we do not know their exact accommodation. (*FGD15_VMW_Male).

76.7 % of MMWs and VMWs in Zone 1, which contains a higher number of remote villages, and 75.6 % in Zone 2, expressed major difficulties posed by long-distance travel. Because VMWs and MMWs found it so difficult to reach out to MMPs themselves, they frequently requested that village chiefs take on this role.

#### Management of health resources

Most VMWs and MMWs reported following correct storage practices for medications (81.2 %) and rapid diagnostic tests (RDTs) (79.7 %). When asked about stock outs in the last 3 months, 57.4 % reported that RDTs had been out of stock for more than a week, and anti-malarials for even greater periods of time. The interviewees stated that 84.3 % of stock outs were caused by a lack of supply from the HC, and 61.2 % were caused by unexpected demand. In the qualitative interviews, workers discussed how during stock-outs, patients experience interrupted or delayed treatment before the VMW/MMW’s monthly restocking trip. In some instances, patients were initially referred to the HC. Nevertheless the HCs are far, and have restricted opening hours, which coincide with the patients’ own working hours. Many patients reported that they bypassed the HC for this reason, and went directly to the private sector. Patients who lacked the funds to do this often used traditional medicine. Some VMWs, out of concern, used their own resources to help patients travel to the HC. Stock-outs make adherence by workers to the national treatment guidelines difficult or impossible; some discussed using other anti-malarial treatments available in the community in order to provide some care:

*Sometimes we have to buy anti*-*malarials from private clinics, as we are out of stock. In this case we have to charge the patient, and treatment is no longer free of charge, as they mentioned to us upon recruitment. We get blamed by patients, as they have to spend money for treatment.* (FGD43_VMW_Female).

Stock outs impact more than quality of care. Community members expressed dissatisfaction at having to go to the HC for anti-malarials or tests during stock outs. VMWs/MMWs expressed concern that these shortages erode the community’s trust in them, which then results in reduced patient motivation to access their services.

### Job satisfaction

#### Motivating factors

VMWs and MMWs reported being highly motivated to perform their job as a volunteer, and being committed to the aim of malaria elimination in Cambodia. Motivating factors included the fact that they are making a contribution to malaria control in the community (50.8 %), the social responsibility they feel to help others (31.5 %), and the benefits of attaining new skills and knowledge (17.3 %). Over 90 % reported that they would want to continue to provide malaria services even if the VMW project were to conclude. Respondents also described being motivated because the community showed confidence in the quality of their work. All but one reported that patients accept and trust them to provide good quality malaria services, and that this volunteer job has increased their personal standing in the community.

#### Worker satisfaction

The majority of VMWs/MMWs (84 %) were satisfied that the programme increased community malaria awareness, and all but one respondent felt adequately supported by HC staff. Nevertheless, VMWs and MMWs reported that as malaria infections decline in this region, there are fewer opportunities for them to use their practical skills, which negatively impacted their motivation. Limited access to resources essential to their job, such as petrol, transportation, and tools like satchels or bags, were also said to demotivate. Similarly, respondents lacked sufficient information, education and communication (IEC) materials to support the delivery of key malaria messages, without which they found it difficult to provide effective education. Other factors that appeared to affect their motivation were a lack of supervisory visits and feedback from their line managers, and stock outs.

Trust and appreciation from the community was a strong motivating factor for the workers. Nevertheless, they reported that this is often eroded when a patient presenting with fever and other malaria symptoms tests negative for malaria. Protocol dictates that such patients must be referred to the nearest HC, which is often inaccessible. Patients want and sometimes demand treatment from VMWs/MMWs for other illnesses, and workers are frustrated when they cannot provide it. Due to the reduction in malaria prevalence, this scenario is increasingly common. VMWs stated that their decreased malaria caseloads would allow them to take on other responsibilities, and they felt this would help them maintain trust from the communities they served, and improve their own motivation to work.

## Discussion

This is the first assessment of the VMW programme carried out within catchment areas with confirmed ART-R in Western Cambodia [21]. The assessment found, overall, competent job performance compared to the workers’ benchmarked indicators (above 80 %) according to their terms of reference. Nevertheless, the elimination of falciparum malaria will require 100 % performance, as cases of untreated or incompletely treated malaria are important factors in the development of anti-malarial resistance [22, 23]. For this reason, each and every case that the VMW programme is unable to treat successfully, whether due to organizational or health system failures, or dwindling worker motivation, is potentially exacerbating the spread of ART-R, and therefore compromising malaria elimination. Studies have shown that in other malaria-endemic countries, CHWs face similar system deficiencies to those identified in this study, and that these significantly interfere with the effectiveness of malaria interventions [24–27]. In order to improve the performance of Cambodia’s VMW network, it is recommended that the following areas be addressed:

### Worker malaria knowledge

The study found that, overall, the majority of VMWs and MMWs correctly understand malaria transmission. Nevertheless there is a sizeable minority of workers who confuse malaria prevention methods with the prevention of other health issues, a situation that has also been observed in Ethiopia, Tanzania and Bangladesh [28–33]. Given the high level of trust that communities place in their VMWs/MMWs, addressing these misconceptions is crucial to prevent them from spreading further. The VMW network should be re-trained on key health education messages.

### Adherence to DOT guidelines

DOT is a critical component of the Cambodian strategy for the containment of ART-R parasites [13]. VMWs and MMWs reported high adherence to DOT for 1st day doses, which was drastically lowered for 2nd and 3rd day doses. This was mostly attributed by workers to a lack of transportation that would enable them to visit patients, and to the difficulties in locating highly mobile patients. This study found that VMWs and MMWs tend to have a good understanding of malaria resistance and its relationship to incomplete treatment regimens. Therefore, it seems that the barriers to DOT implementation are largely logistical, not knowledge-related. Workers should be provided with the means to access remote patients; otherwise, adherence to DOT is unlikely to improve despite increased behaviour change communication (BCC) in the area.

Workers believe that DOT cannot be implemented among MMPs due to their high mobility, and requested in this study that village chiefs, who have better access to MMPs, be the ones to reach out to them. Alternatives to DOT that can ensure patient compliance among MMPs should also be explored through operational research. Two randomized controlled trials recently conducted in Kenya showed that text message reminders can improve adherence to treatment [34, 35].

### Transportation

Aside from VMWs and MMWs themselves, transportation difficulties also affect patients trying to access health services. Innovative transportation schemes accessible by all affected parties should be considered. Operational research may shed light on how best to address this problem, as has been suggested by the Cambodian Research Consortium [36]. Most of the current research on innovative transportation comes from maternal and child health programmes in Africa; there is little evidence regarding South-East Asia.

### Stock-outs

Stock-outs can lead to both workers and patients relying on the private sector, which is particularly worrisome. Using private sector malaria medications is against the national guidelines. A recent study found that in Western Cambodia most private sector anti-malarials are either substandard or counterfeit [37]. The national programme has piloted an m-Health system to address supply chain issues and it is now implementing it across the country. It was found to increase access to artemisinin-based combination therapy and RDTs in the public sector, which in turn led to a decrease in malaria morbidity and mortality [38]. Several factors may be responsible for the stock-outs. Previous research in Cambodia has shown that VMWs tend to run out of RDTs and anti-malarials more rapidly during the rainy season, due to a higher demand, or to difficulties in replenishing supplies (heavy workload in the fields, weather-related inaccessibility) [39]. Previous studies have also warned that supply chain strengthening in Cambodia, including forecasting and monitoring, is vital for VMWs to be effective [40, 41]. Treatment delays and interruptions in an ART-R context can be very detrimental to patients’ prognoses and strengthen parasite resistance. In order to address supply chain weaknesses, bottlenecks will need to be clearly identified in different geographical areas within each zone, and addressed.

### Expansion of the VMW role

According to this study, VMWs, MMWs, as well as community members at large, want malaria workers to diagnose and treat other diseases too. Studies in various elimination settings confirm that as malaria infections become rarer, the VMW network will increasingly need to be able to diagnose, treat, and prevent other common diseases in order to be seen as adding value to communities, and maintain worker motivation [42]. A community health worker programme integrated into the primary healthcare system can promote care at the household level and function as a crucial link between community members and the primary healthcare care system. In Cambodia, a 2008 pilot project in which VMWs provided additional health services resulted in more frequent access of VMW services by community members, and increased worker motivation. Capitalizing on this success, the role of VMWs and MMWs in Cambodia should be expanded to include acute respiratory infections and diarrhoea in children under five.

### Reaching mobile and migrant populations

Determining how best to meet the needs of mobile populations should be prioritized in the national research agenda. Respondents to this study indicated that migrant workers often contract malaria shortly after their arrival because they are unfamiliar with prevention methods. They also indicated that migrants are extremely difficult to treat effectively. This is consistent with a recent survey by URC, which shows that most migrants travel from malaria-free to endemic provinces [43]. Pre-departure education, which was widely advocated by study respondents, will therefore be vital to malaria elimination. Previous efforts to implement it have had very limited success, as it is difficult to determine which individuals are about to travel and should be targeted. Pre-departure education should therefore be combined with on-the-way education, which has also been piloted but not evaluated. Several respondent-driven sampling and applied anthropological studies on the Cambodia and Thailand borders [44–46] have indicated that there are significant variances in human population movement (HPM), showing the need for differential malaria control and elimination strategies within and across contexts in order to target different mobile populations [47, 48].

### More community health workers

There is wide consensus that VMW coverage is lacking nationwide. The national programme is currently expanding the number of VMW villages in Cambodia, which will contribute significantly to malaria elimination efforts. Nevertheless, the VMW network should be extended to all communities beyond reasonable reach of health facilities nation-wide [12], particularly if the scope of VMW services is expanded. In addition to addressing serious health concerns like acute respiratory infections, this will help to mitigate problems caused by long distances and lack of transportation, increase the availability of VMWs for consultation during peak hours, and provide pre-departure malaria education in non-endemic areas.

### Considerations when scaling up

It is clear that other countries have benefitted from the integration of malaria integrated community case management (ICCM) with other health services. Important facilitating factors for success include interactive and practical training, clear guidelines, and regular supportive supervision [49]. Nevertheless, scaling up VMWs’ expanded roles to a national level depends on the health system’s capacity and readiness. Other national programmes that have reported poor supervision, variable quality of care, and stock outs of supplies and equipment, have been unable to implement adequate clinical supervision [50–53] in expanded programmes. At the same time, some CHW programmes have been scaled up too quickly, seriously undermining their quality and effectiveness [54, 55]. Furthermore, little is known about ICCM implementation in ART-R containment settings, and there is an urgent need for more data regarding worker supervision in scaled programmes, as well as the most effective method for referring patients to HCs when malaria workers cannot treat them [49]. The national programme should consider these critical aspects before scaling up interventions. Brazil, Bangladesh and Nepal successfully scaled up their programmes gradually and deliberately, with adjustments made along the way [56].

### Worker motivation

The impact of community health worker motivation on job performance is rarely explored [57–60]. Nevertheless, recent studies have shown that it plays a significant role [61–63]. This study found that VMW/MMW motivation is generally high, and efforts, discussed above, to reduce challenges and expand the VMW programme will help to maintain and improve it. Nevertheless, other demotivating factors should be eliminated. The lack of supplies necessary for day-to-day activities, like bags and IEC materials, must be addressed at the system level. More frequent performance management that focuses on showing appreciation can address the demotivating systemic lack of feedback from supervisors. Incentives such as training and career development, as well as mechanisms that allow workers to receive regular feedback from the community, can bolster respect in the community and improve worker motivation.

## Study limitations

Like any survey, the results from the survey were based on what the participants had provided, which is difficult to really contrast what they said with their actual behaviour of the participants. The results only refer to HCs with 10 or more VMWs/MMWs. There could be systematic differences in performance and practice of those VMWs and MMWs working in HCs of smaller size due to, for instance, differences in training and supervision, and this deserves further evaluation.

## Conclusions

Malaria control and elimination depend on high-performance levels in the health system. This high performance means delivering malaria interventions at high and equitable levels of quality, and with effective universal coverage. This will require providing geographical access to target populations, ensuring consistent high-standard care, and securing the motivation of malaria workers, as well as their logistical ability to provide services. It is important to have strong evaluation elements as the programme rolls out, in order to identify emerging challenges and obstacles, and to design appropriate measurable responses to these findings [64].

## Abbreviations

ART-R: artemisinin resistant
ASEAN: Association of Southeast Asian Nations
BCC: behaviour change communication
CHW: community health worker
CNM: National Programme for parasitology, entomology and malaria control (Cambodia)
DOT: directly-observed treatment
FGD: focus group discussion
HC: health centre
ICCM: integrated community case management
IDI: in-depth interview
IEC: information, education and communication
MMP: mobile and migrant population
MMW: mobile malaria worker
OD: operational district
RDT: rapid diagnostic test
VMW: village malaria worker
WHO: World Health Organization
